# Educational Status, Cancer Stage, and Survival in South India: A Population-Based Study

**DOI:** 10.1200/GO.20.00259

**Published:** 2020-11-06

**Authors:** Aleyamma Mathew, Preethi Sara George, Ramadas Kunnambath, Beela Sarah Mathew, Aswin Kumar, Roshni Syampramod, Christopher M. Booth

**Affiliations:** ^1^ Department of Cancer Epidemiology, Queen’s University, Kingston, Ontario, Canada; ^2^ Department of Radiation Oncology, Queen’s University, Kingston, Ontario, Canada; ^3^ Regional Cancer Centre, Trivandrum, Kerala, India; ^4^ Department, of Oncology, Queen’s University, Kingston, Ontario, Canada; ^5^ Department of Public Health Sciences, Queen’s University, Kingston, Ontario, Canada

## Abstract

**PURPOSE:**

Lower socioeconomic status is associated with more advanced cancer at the time of cancer diagnosis. It is unknown whether this leads to inferior survival in low- and middle-income countries. Here, we explore the association between educational level and survival in South India.

**METHODS:**

The Trivandrum Cancer Registry (3.3 million population) was used to identify all cases of breast and cervical cancer (women) and oral cavity (OC) and lung cancer (men) diagnosed during 2012-2014. Educational level was classified as illiterate/primary school, middle school, and secondary school and above. Survival was measured from date of diagnosis using the Kaplan-Meier method. Cox proportional hazards regression modeling was used to describe the associations among education, stage of cancer at diagnosis, and survival.

**RESULTS:**

The study population included 3,640 patients with breast (n = 1,727), cervical (n = 425), OC (n = 702), and lung (n = 786) cancer. Educational level was 27%, 23%, and 32% for illiterate/primary, middle, and secondary school and above, respectively. The 5-year survival rate for breast cancer was 59%, 68%, and 73% (*P* = .001); for cervical cancer, 51%, 52%, and 60% (*P* = .146); and for OC cancer, 42%, 35%, and 48% (*P* = .012) for illiterate/primary, middle school, and secondary school and above, respectively. The survival gradient across social groups was substantially attenuated when stage was added to the multivariable model. There was no observed difference in survival across educational groups for lung cancer (2%, 4%, and 3%; *P* = .224).

**CONCLUSION:**

Data from this population-based study in South India demonstrate that patients from a lower educational background have inferior survival and that this is at least partially explained by having more advanced disease at the time of diagnosis. Public health efforts are needed to facilitate timely diagnosis and reduce disparities in cancer outcomes.

## INTRODUCTION

In high-income countries (HICs), it is known that lower socioeconomic status (SES) is associated with inferior survival.^1-4^ Potential mechanisms to explain survival differences between social groups include differences in tumor biology, patient comorbidity, stage of disease at diagnosis, access to therapy, and treatment practices. The pivotal systematic review by Woods et al^2^ demonstrated inferior cancer survival among adults in low SES groups in HICs; there were no studies from low- and middle-income countries (LMICs). In a systematic review of pediatric cancer, Gupta et al^5^ did find evidence of a survival gradient across social groups in LMICs. We are not aware of any studies that have described the extent to which SES is associated with cancer outcomes in adults living in LMICs.

**CONTEXT**

**Key Objective**
To understand if socioeconomic status is associated with cancer survival in the Indian context and to what extent this is explained by differences in stage of cancer at diagnosis.
**Knowledge Generated**
Patients with low education status (illiterate/primary school) had inferior survival for breast, cervical, and oral cavity cancers compared with those with secondary school education. This survival gradient was attenuated when stage at diagnosis was added to the multivariable model.
**Relevance**
Patients from lower educational background have inferior cancer survival. Public health efforts are needed to facilitate timely diagnosis and reduce disparities in cancer outcomes.

We have recently reported a population-based study from South India that demonstrated a strong association between lower educational status and more advanced cancer at time of diagnosis.^6^ Our study included breast and cervical cancer in women and lung and oral cavity cancer in men because they represent the highest burden of cancer in India. In the current study, we present survival data for the same cohort of patients. The objectives of the current study were to describe whether there is an association between educational status and cancer survival and explore the extent to which differences in stage of disease at diagnosis might explain this observed association.

## METHODS

### Study Design and Population

This is a retrospective population-based cohort study. The study was approved by the institutional review board at the Regional Cancer Centre (RCC), Trivandrum. The Population-Based Cancer Registry (PBCR) of Trivandrum District in Kerala, India, was used to identify all incident cases of breast and cervical cancer in women and oral cavity and lung cancer in men diagnosed during 2012-2014. The Trivandrum District has a population of 3.3 million (54% urban and 46% rural). The State of Kerala (population 33 million) is located in South India and has the highest life expectancy (74 *v* 64 years), lowest infant mortality rate, and highest literacy rate (94% *v* 73% national rate) in India.^7^

### Data Sources

The Trivandrum PBCR is one of 27 cancer registries that operate under the National Cancer Registry Program of India. The PBCR uses an active case finding methodology by visiting government/private hospitals and pathology laboratories. Data have been collected from > 60 hospitals and seven pathology laboratories. Computerized information processing includes linkage of patient data obtained from various sources and review of duplicate/redundant records. The validity of data is monitored by conducting data quality exercises periodically on abstraction of data from medical records and coding of the diagnosis. Microscopic confirmation, death certificate only, fatality ratio (%), and proportion of unknown primary sites are used to assess the quality of the registry.

The major sources for incidence data are the RCC (physical location of the registry; 63% of cases) and the Government Medical College Hospital (MCH; 24% of cases), both located in Trivandrum. A large number of private hospitals (n = 47) and government hospitals (n = 32) also diagnose and treat patients with cancer. Because cancer is not a notifiable disease in India, registration of incident cancer cases is carried out by active case finding. Based on an administrative letter provided by the principal secretary, Government of Kerala, to all health authorities in the district, cooperation from all hospitals has been obtained. The Trivandrum PBCR employs 14 tumor registrars trained in cancer registration through locally and nationally organized courses followed by continuing in-service training. PBCR staff review medical records from 60 potential data sources and seven pathology laboratories at regular intervals to abstract data on incident cancer cases. The information collected includes age, residential address, sex, religion, marital status, education, date of incidence, basis of diagnosis, topography, morphology, clinical extent of disease, treatment details, and vital status.

### Definitions of Exposures and Outcomes

Sociodemographic characteristics are captured routinely by PBCR staff. In this study, we explored the association among survival, stage of disease, and education. Education was chosen as a surrogate measure of SES. Information on household income was not considered in this study because of high rates of missing data and concerns about the validity of self-reported income. Self-reported educational levels were illiterate, up to primary school, up to middle school, up to secondary school, college, and technical school. In the current analysis, patients were classified into three groups: illiterate/primary (0-5 years), middle school (6-10 years), and secondary school and above (> 10 years).

Stage at diagnosis is routinely assigned by the treating oncologist for all cases seen at RCC and Government MCH. If clinical stage is not available, pathologic stage is recorded. Staging systems vary by disease site: Breast and oral cavity cancer are staged using the TNM classification system, cervical cancer is staged using the International Federation of Gynecology and Obstetrics system, and lung cancer is staged using the SEER clinical extent of disease system.

In cases of missing data, PBCR staff reviewed primary data sources to assign stage of disease and contacted patients by telephone to ascertain educational status. Among a random sample of 10% of cases, the agreement rate between oncologist-assigned stage and PBCR stage grouping was high: 92% for breast, 91% for cervical, 89% for oral cavity, and 88% for lung cancer. The primary end point of the study was to evaluate the association among educational level, stage of cancer at diagnosis, and survival.

### Statistical Analysis

Overall survival was measured from date of diagnosis using the Kaplan-Meier method; summary measures are reported as hazard ratios (HRs). The Cox proportional hazards model was used to describe the associations among educational level, stage of cancer at diagnosis, and survival; these variables were selected a priori.

## RESULTS

### Characteristics of Study Population

The overall population included 4,547 patients with breast (n = 2,283), cervical (n = 481), lung (n = 986), and oral cavity (n = 797) cancer diagnosed in Trivandrum District during 2012-2014. A total of 2,303 (50%) died during the 5-year follow-up period, > 5-year follow-up information is available for 3,640 patients (78%) and < 5-year for 664 patients (14%), and 351 patients (8%) had no follow-up (Appendix Table A1). Characteristics of the study population are listed in Table 1. Differences between patients with and without follow-up data are listed in Table 2. Patients without follow-up data (and thereby excluded from the current analysis) were more likely to be treated at peripheral centers and to have missing stage/educational status data. Mean age of the study population was 58 years, and 31% were ≥ 65 years of age. Eighty-four percent of patients were married. The majority (73%) of patients were Hindu, and 32% had attended secondary school. Within the four cancer subgroups, patients with breast cancer were younger and more highly educated. A lower proportion of women with cervical cancer were married relative to the other cancers. Seventy-nine percent of patients (2,861 of 3,640) were identified from RCC, 15% (547 of 3,640) from Government MCH, and 6% (232 of 3,640) from other centers.

**TABLE 1:**
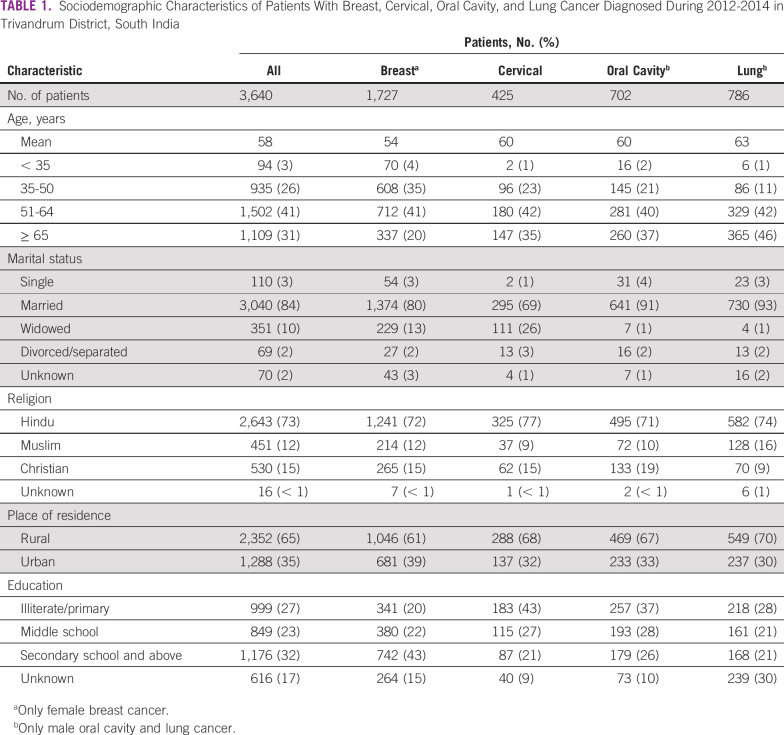
Sociodemographic Characteristics of Patients With Breast, Cervical, Oral Cavity, and Lung Cancer Diagnosed During 2012-2014 in Trivandrum District, South India

**TABLE 2:**
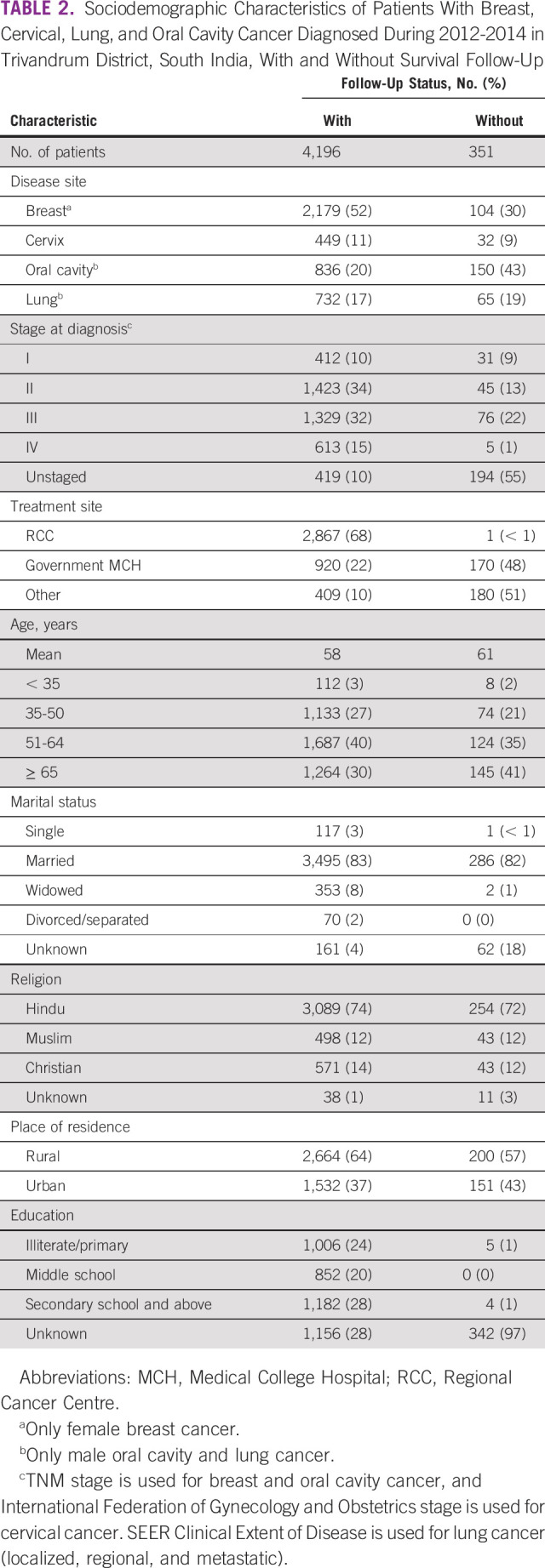
Sociodemographic Characteristics of Patients With Breast, Cervical, Lung, and Oral Cavity Cancer Diagnosed During 2012-2014 in Trivandrum District, South India, With and Without Survival Follow-Up

### Survival, Educational Status, and Stage of Disease

**FIG 1:**
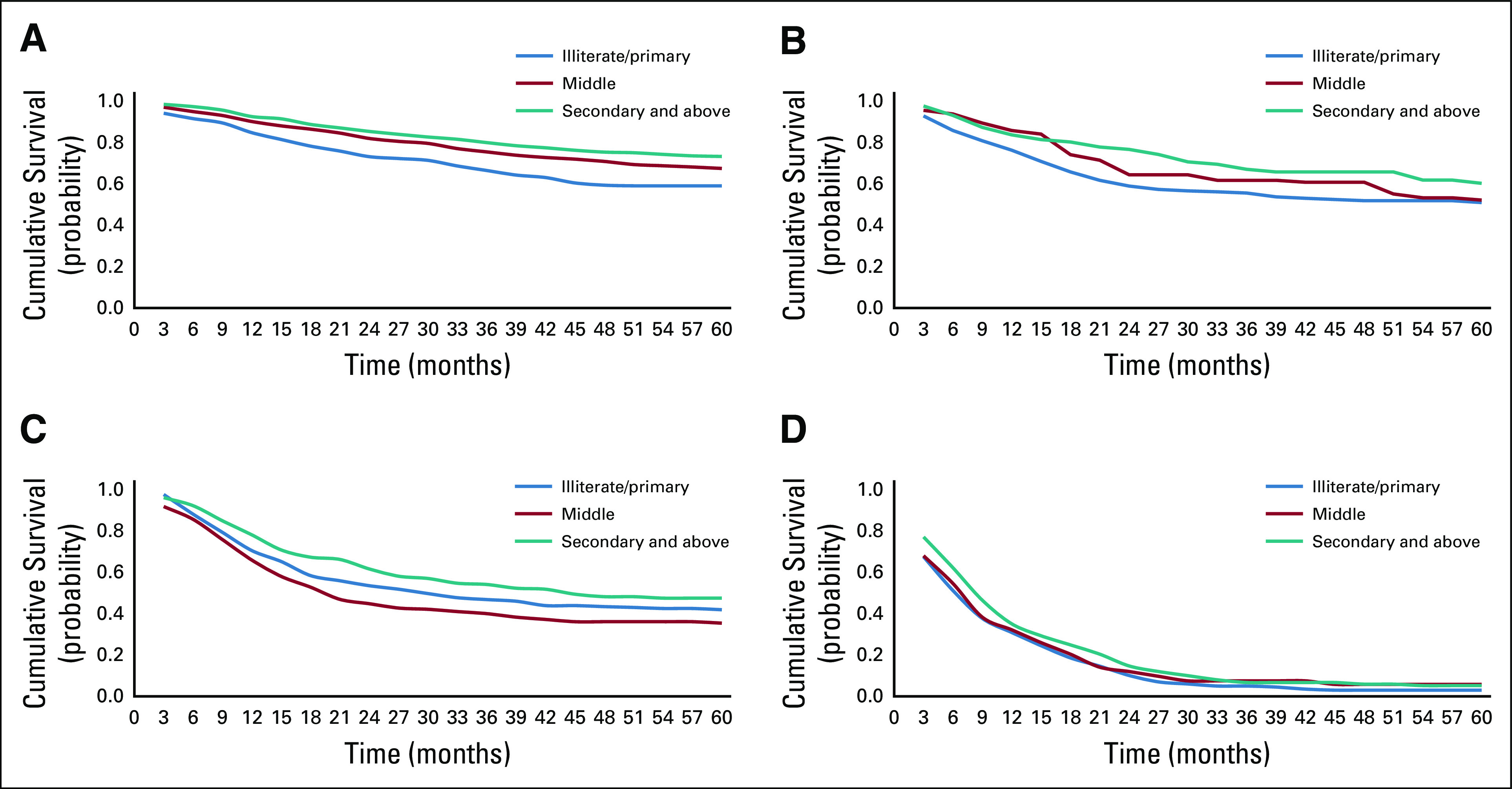
Survival stratified by educational status of patients with breast, cervical, oral cavity, and lung cancer diagnosed in Trivandrum District during 2012-2014. (A) Breast cancer (female). (B) Cervical-uterine cancer. (C) Male oral cavity. (D) Male lung cancer.

Survival of patients by educational status is listed in Table 3 and shown in Figure 1. There is a clear stepwise gradient in survival across educational groups among patients with breast cancer. The 5-year survival rate was 59%, 68%, and 73% (*P* = .001) for the illiterate/primary, middle school, and secondary school and above groups, respectively. Similar results were found in oral cavity cancer, with survival rates by educational level of 42%, 35%, and 48% (*P* = .012), respectively. A comparable trend was seen in cervical cancer (51%, 52%, and 60%, respectively), although the observed differences in these smaller cohorts were not statistically significant (*P* = .146). There was no discernable difference in outcome across educational groups for patients with lung cancer (3%, 6%, and 5%, respectively; *P* = .154).

**TABLE 3:**
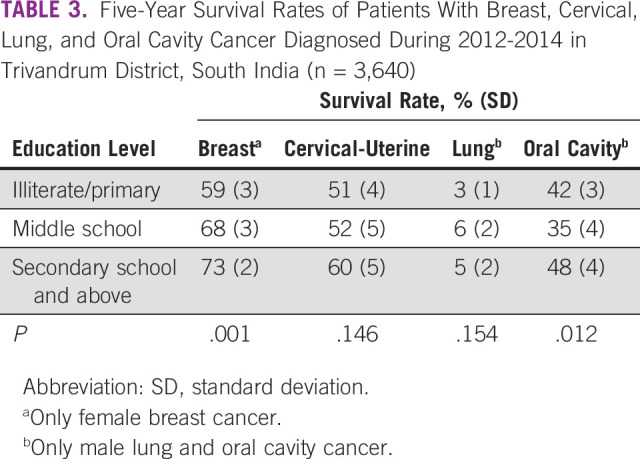
Five-Year Survival Rates of Patients With Breast, Cervical, Lung, and Oral Cavity Cancer Diagnosed During 2012-2014 in Trivandrum District, South India (n = 3,640)

Multivariable analyses that adjusted for age and stage are listed in Table 4. When stage was added to the model, there was a substantial attenuation of effect for breast (unadjusted HR, 1.7 [95% CI, 1.4 to 2.1]; adjusted HR, 1.5 [95% CI, 1.2 to 1.9]), cervical (unadjusted HR, 1.5 [95% CI, 1.0 to 2.2]; adjusted HR, 1.1 [95% CI, 0.7 to 1.7]), and oral cavity (unadjusted HR, 1.2 [95% CI, 0.9 to 1.5]; adjusted HR, 0.9 [95% CI, 0.7 to 1.20]) cancer. There was no evidence of collinearity in the model.

**TABLE 4:**
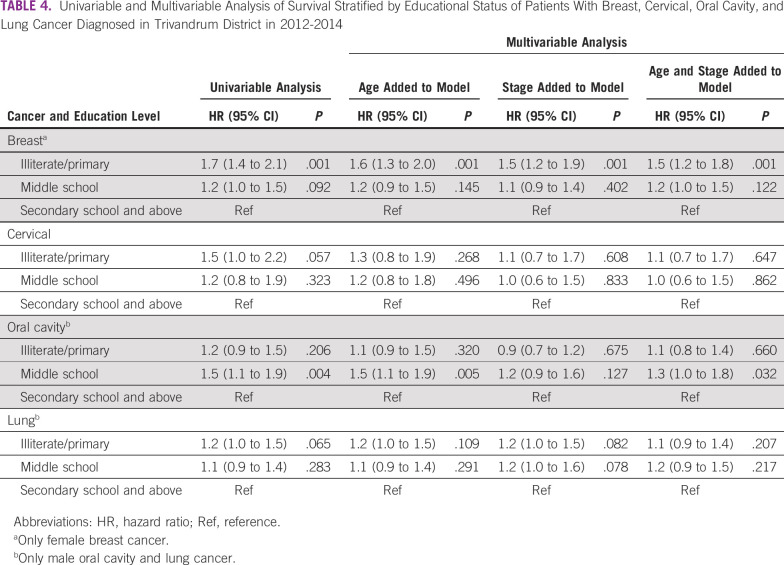
Univariable and Multivariable Analysis of Survival Stratified by Educational Status of Patients With Breast, Cervical, Oral Cavity, and Lung Cancer Diagnosed in Trivandrum District in 2012-2014

## DISCUSSION

In this study, we describe survival of patients with cancer in the general population of South India and explore the extent to which survival is associated with educational status. Our study focused on the four most common cancers in India. Several important findings have emerged. First, our results demonstrate that survival of patients in India is far lower than outcomes reported in HICs. Second, our study demonstrates a strong association between educational status and survival of breast cancer and oral cavity cancer; a comparable trend is also seen for cervical cancer. Finally, a substantial proportion of the survival gradient across educational groups is explained by differences in stage of cancer at diagnosis. These findings have important policy and public health implications.

It is worth comparing survival observed in our study with population-level reports from other jurisdictions. In the current study, the 5-year survival rate of breast cancer was 54%-70%; this is substantially lower than outcomes reported in the United States, United Kingdom, and Canada, where the 5-year survival rate is 86%-90%.^8-10^ The observed results are comparable to outcomes from other regions of India, where survival is approximately 66%.^11^ Similarly, the 5-year survival rate of cervical cancer in Trivandrum District (44%-55%) is lower than reports from HICs and data from elsewhere in India, where survival is approximately 70% and 59%, respectively.^11^ The survival rate of oral cavity cancer in Trivandrum District is 32%-42%, while it is 60%, 63%, and 65% in the United Kingdom, Canada, and United States, respectively.^8-10^ Finally, the lung cancer survival rate in our current study (2%-4%) is substantially lower than global rates of 10%-20%; survival of 20%-30% is reported by some HICs.^8^

We have previously described an association between low educational status and advanced cancer (stage III-IV) at diagnosis for breast, cervical, and oral cavity cancers in Trivandrum District.^6^ Among 4,547 patients, we found that those with illiterate/primary school education were more likely to have advanced breast (50%, 39%, and 36%; *P* < .001), cervical (46%, 43%, and 24%; *P* = .002), and oral cavity (77%, 76%, and 59%; *P* < .001) cancers compared with patients with higher educational levels, respectively. This association was not seen in lung cancer.

Our results have direct policy relevance. It is increasingly clear that cancer systems in LMICs need to promote earlier diagnosis to improve survival at the population level. Our results suggest that public health educational initiatives should be particularly targeted for populations of lower SES/education. Efforts are needed to promote health literacy, cancer awareness, and access to care. These efforts will be necessary to shift the stage distribution in South India (and other jurisdictions), where the majority of patients are diagnosed with cancer at a very advanced stage.

The primary objective of our current study was to evaluate the extent to which cancer survival was associated with educational status, which was chosen as a surrogate for SES. Studies in HICs commonly used neighborhood median household income from census data as a measure for SES.^4,12,13^ Comparable data in South India were lacking, and we had concerns about the validity of self-reported household income. We therefore used educational level as a surrogate for SES. Work by other groups has demonstrated that educational level in India is correlated with other measures of SES, including occupation, housing, and social status.^14^

Several other studies in India have consistently identified lower education as a risk factor for being diagnosed with more advanced disease.^15-18^ This association was also reported in a systematic review of barriers to breast cancer care in LMICs.^19^ We are not aware of any studies from LMICs that have explored the association between SES and cancer survival in adults. In a pivotal systematic review of this topic, Woods et al^2^ found that most studies reported an association between SES and survival; none of the included studies came from LMICs. Gupta et al^5^ explored the association between SES and survival in a systematic review of pediatric cancers; 36 studies were included of which 10 were from LMICs. In their study, Gupta et al observed a consistent finding that patients from lower SES groups had inferior survival; reasons for this association were not apparent in the published literature.

In their systematic review of studies in HICs, Woods et al^2^ concluded that stage of disease at diagnosis and access to optimal treatment explain a portion of disparity in survival of patients with cancer. We are not aware of any adult studies in LMICs that have explored the association among stage, SES, and survival. The results presented in this study represent the first step in doing so. Our data suggest that a substantial proportion of disparities in survival in South India are explained by differences in stage of cancer at diagnosis. These findings highlight one of the fundamental challenges in India’s cancer system: Most patients are diagnosed with advanced disease. The results of our current study suggest that access to care is the fundamental barrier to timely diagnosis and that this varies across socioeconomic groups. There are a multitude of potential drivers of delayed diagnosis, including poor health literacy, diagnostic nihilism, sociocultural taboos, and economic barriers to care. Although most cancer care is delivered free of charge to patients in the public hospitals of Kerala, additional costs related to travel, accommodation, and lost wages may contribute to differences in treatment patterns among SES groups. Additional research will need to explore public health initiatives that can improve diagnostic accessibility, particularly as it pertains to the most vulnerable populations.

Our study does have methodological limitations that warrant comment. As shown in Appendix Table A1, survival follow-up data were more likely to be missing among patients treated at other institutions compared with the RCC, Trivandrum. This may limit the generalizability of our study results. Moreover, our study cannot fully explain why patients with lower educational status have inferior survival. It is likely that this reflects more aggressive disease and differential cancer treatment, but future work will be needed to explore this more fully to use this knowledge to improve care of patients from impoverished backgrounds.

There are prominent disparities in global cancer care between HICs and LMICs. Like in HICs, it is becoming increasingly clear that socioeconomic disparities also exist within LMICs. As coordinated cancer systems continue to develop in LMICs, it is imperative to understand gaps in care and to identify vulnerable populations that may benefit from focused public health interventions. Data from this population-based study in South India has established that patients from lower educational background are more likely to be diagnosed with advanced-stage cancer and are more likely to have inferior survival. Future work will explore the mechanisms behind these observations in an effort to identify system-level factors that could be modified to reduce disparities in cancer care.

## Figures and Tables

**TABLE A1:**
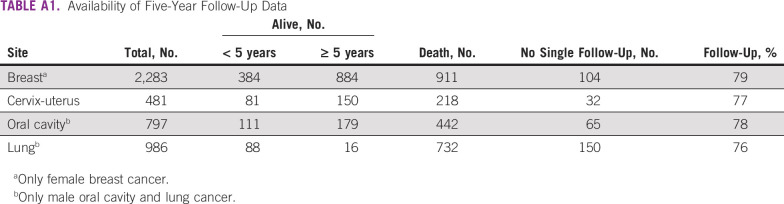
Availability of Five-Year Follow-Up Data
